# Acute Myocardial Infarction in Sub-Saharan Africa: The Need for Data

**DOI:** 10.1371/journal.pone.0096688

**Published:** 2014-05-09

**Authors:** Julian T. Hertz, Joseph M. Reardon, Clarissa G. Rodrigues, Luciano de Andrade, Alexander T. Limkakeng, Gerald S. Bloomfield, Catherine A. Lynch

**Affiliations:** 1 Department of Emergency Medicine, Vanderbilt University, Nashville, Tennessee, United States of America; 2 Division of Emergency Medicine, Duke University, Durham, North Carolina, United States of America; 3 Division of Cardiology and Duke Clinical Research Institute, Duke University, Durham, North Carolina, United States of America; 4 Global Health Institute, Duke University, Durham, North Carolina, United States of America; 5 Department of Surgery, Duke University, Durham, North Carolina, United States of America; 6 Instituto de Cardiologia do RS – Fundação Universitária de Cardiologia, Porto Alegre, RS, Brazil; 7 Universidade Estadual do Oeste do Paraná (UNIOESTE), Foz do Iguaçú, PR, Brazil; Universidad Peruana Cayetano Heredia, Peru

## Abstract

**Background:**

Trends in the prevalence of acute myocardial infarction in sub-Saharan Africa have not been well described, despite growing recognition of the increasing burden of cardiovascular disease in low- and middle-income countries. The aim of this systematic review was to describe the prevalence of acute myocardial infarction in sub-Saharan Africa.

**Methods:**

We searched PubMed, EMBASE, Global Health Archive, CINAHL, and Web of Science, and conducted reference and citation analyses. Inclusion criteria were: observational studies, studies that reported incidence or prevalence of acute myocardial infarction, studies conducted in sub-Saharan Africa, and studies that defined acute myocardial infarction by EKG changes or elevation of cardiac biomarkers. Studies conducted prior to 1992 were excluded. Two independent reviewers analyzed titles and abstracts, full-texts, and references and citations. These reviewers also performed quality assessment and data extraction. Quality assessment was conducted with a validated scale for observational studies.

**Findings:**

Of 2292 records retrieved, seven studies met all inclusion criteria. These studies included a total of 92,378 participants from highly heterogeneous study populations in five different countries. Methodological quality assessment demonstrated scores ranging from 3 to 7 points (on an 8-point scale). Prevalence of acute myocardial infarction ranged from 0.1 to 10.4% among the included studies.

**Interpretation:**

There is insufficient population-based data describing the prevalence of acute myocardial infarction in sub-Saharan Africa. Well-designed registries and surveillance studies that capture the broad and diverse population with acute myocardial infarction in sub-Saharan Africa using common diagnostic criteria are critical in order to guide prevention and treatment strategies.

**Registration:**

Registered in International Prospective Register of Systematic Reviews (PROSPERO) Database #CRD42012003161.

## Introduction

While ischemic heart disease is a leading cause of disability-adjusted life years (DALYs) lost among wealthy nations, its impact in sub-Saharan Africa (SSA) has historically been limited, due to the relatively high burden of communicable diseases and poverty-associated diseases in these countries.[1], [2] In recent years, however, SSA has experienced a sharp rise in risk factors for ischemic heart disease, including aging of the population, reduced exercise, poor diet, and uncontrolled hypertension.[3]–[5] If such demographic and epidemiologic changes have resulted in an increasing prevalence of ischemic heart disease, the ability of local health ministries and facilities to adequately anticipate and respond to these emerging threats depends on their ability to accurately forecast the current and future burden of disease. As a result, several authors have highlighted the increased need for accurate and timely data regarding the burden of disease of acute myocardial infarction (AMI) in the region.[6]–[8] Although AMI is not the only manifestation of ischemic heart disease, it is one of the most lethal and easily identified sequelae of the disease, and is thus ideally suited for studies in low-resource settings where more costly measurements of ischemic heart disease are not feasible.

A recent global burden of disease study systematic review of AMI incidence worldwide using strict criteria failed to identify any high-quality studies from the sub-Saharan African region.[9] Our systematic review attempted to compensate for the lack of high-quality population-wide data by using expanded inclusion criteria encompassing studies from sub-populations within SSA. It is unclear whether the absence of high-quality data regarding the incidence or prevalence of AMI in the region is due to low prevalence of AMI or a lack of research interest and capacity.

Despite the growing recognition of the need for a better understanding of the prevalence of AMI in SSA, no study has yet compiled existing data into a single resource to compare rates across settings or time. Because high-quality population-based studies of the prevalence of AMI have not been performed, we sought to aggregate published data regarding the prevalence of AMI among any sub-populations in SSA in order to shed light on the burden of this disease in the region. To that end, we conducted a systematic review to assess the prevalence of AMI in SSA and identified seven studies that report the prevalence of AMI in a sub-population, with variable levels of methodological rigor.

## Methods

### Ethics statement

This systematic review did not require human subjects or institutional review board oversight. It is reported in accordance with PRISMA guidelines and is registered in the PROSPERO (International Prospective Register of Systematic Reviews) database (Located at http://www.crd.york.ac.uk/NIHR_PROSPERO/) (Registration #CRD42012003161).[10], [11]

### Eligibility criteria

Observational studies were included that reported the incidence or prevalence of AMI among any population within SSA. Published articles were required to be in English, Spanish, French or Portuguese, or have complete translations into one of those languages. The study definition of “acute myocardial infarction” was based on the universal definition of myocardial infarction, defined by a combination of clinical symptoms felt by the clinician to represent coronary ischemia within 24 hours of presentation to the hospital or during the present hospitalization and either EKG changes (new ST-segment elevation, new LBBB, new pathologic Q wave) or elevation of cardiac enzymes (troponin or CK-MB >99th percentile) with or without ST-segment elevations on EKG.[12] Our study definition did not require biomarker elevations as most studies were conducted in areas without access to these tests. In situations where more than one study was conducted on the same data set, the study with more complete data was included. Studies were included from January 1992 through October 28, 2012.

### Information sources

The following electronic databases were searched: PubMed, Embase, Global Health Archive, CINAHL, and Web of Science. In addition, two independent reviewers (JTH and JMR) manually evaluated the references of the included articles and performed a citation analysis of the included studies using Google Scholar. Additional proposed articles for inclusion were solicited from the authors of the included studies through email communications. These information sources will be made available to interested readers upon email request to the authors.

### Search

The initial search comprised the MeSH terms “Africa”, “Africa South of the Sahara”, “Africa, Central”, “Africa, Eastern”, “Africa, Western”, “Africa, Southern”, “Chest Pain”, “Acute Coronary Syndrome”, “Myocardial Ischemia”, “Angina Pectoris”, “Angina, Unstable”, “Myocardial Infarction” and related entry terms. The complete search strategy used for the PubMed database is shown in Appendix S1. Limits for language and/or time were not used in the initial search.

### Study selection

Titles and abstracts of the retrieved articles were independently evaluated by 2 reviewers (JTH and JMR), and assessed for eligibility according to inclusion criteria. Any abstract considered by either reviewer to potentially be eligible for inclusion was then subjected to a full-text evaluation by both reviewers independently to determine eligibility and documented in separate spreadsheets. Disagreements regarding study eligibility were resolved by consensus and re-review, and if disagreements persisted, a third reviewer decided (CAL).

### Quality and bias of studies

The quality of the included studies was assessed using the Loney scoring system for evaluating incidence and prevalence studies.[13] The Loney system is based on the presence (score of 1) or absence (score of 0) of the following criteria: (1) random sample or whole population under study, (2) unbiased sampling frame or list from which subjects are drawn, (3) adequate sample size (>300 subjects), (4) standardised measures, (5) outcomes measured by unbiased (blinded) assessors, (6) adequate response rate (at least 70%), (7) confidence intervals (CI), subgroup analysis, and description of refusers provided and (8) all study subjects described. The maximum score was 8 points. Scores of 0 to 4 indicate lower quality, while scores greater than 5 suggest higher quality.

### Data extraction

Two reviewers (JTH and JMR) independently conducted the data extraction and any disagreements in the extracted data were resolved by the third reviewer (CAL). The recorded characteristics of the studies included study design, measured prevalence of AMI, and any covariates measured with MI (lifestyle risk factors, patient demographics, and comorbid diseases).

### Data analysis

A descriptive analysis of the included studies was performed. AMI prevalence for each study was calculated by dividing the number of patients diagnosed with AMI by the total number of patients evaluated in the study. Since studies represented highly heterogeneous populations, a meta-analysis was not performed. Formal GIS maps were constructed to show the distribution of studies across SSA. Data were processed using the open sourced software R Language version 3.0.1 and Quantum GIS (QGIS) version 1.9.0-Master.[14], [15]

## Results

### Study selection

**Figure 1:**
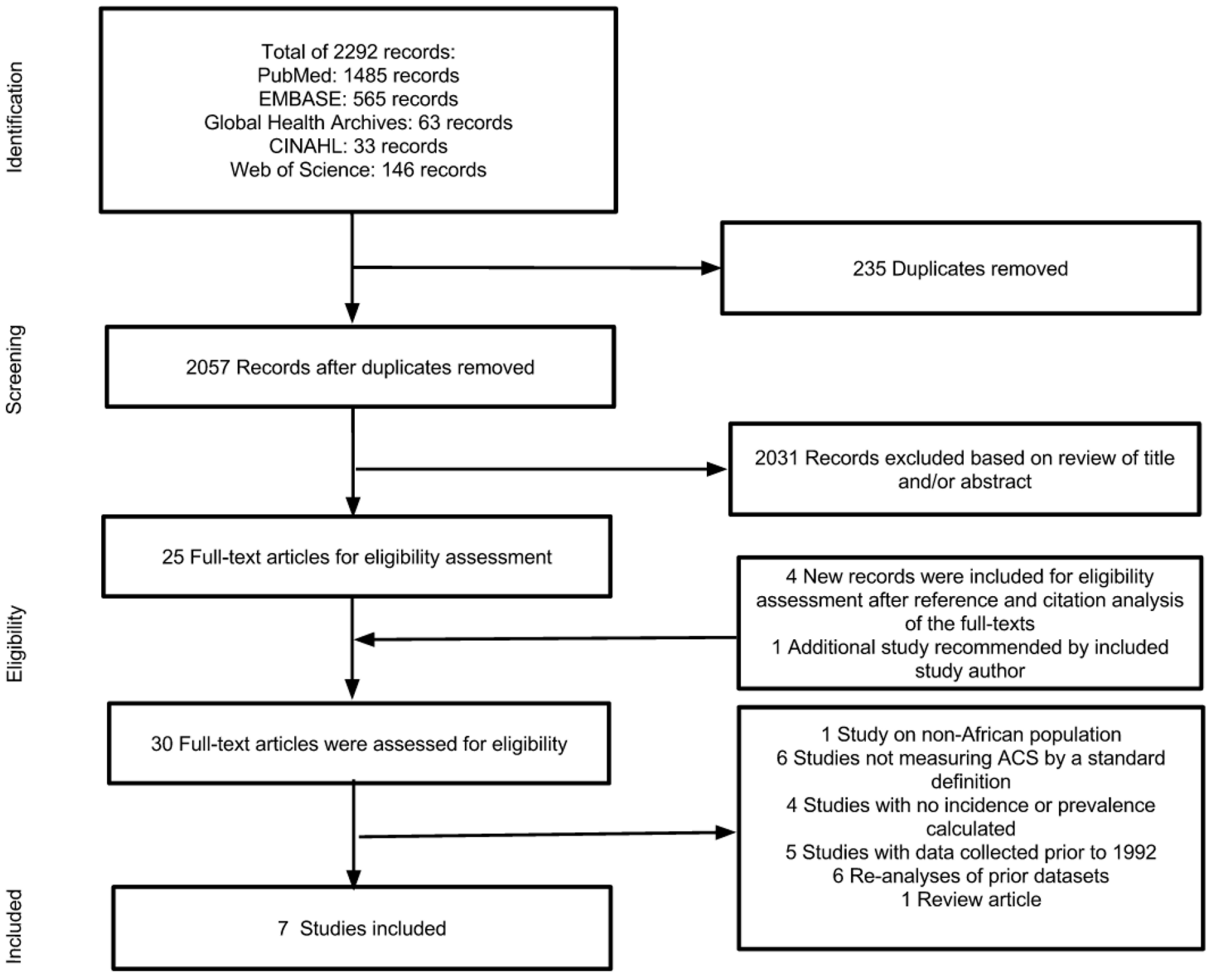
Study flow diagram.

A total of 2292 records were retrieved from the databases, 29 of which were selected for full-text assessment of eligibility. Six observational studies from the databases met all criteria. One additional study was proposed by the corresponding author of an included study, and was included.[16] Ultimately, seven studies were included in the systematic review, representing 92,378 patients.[16]–[22] Figure 1 demonstrates the search and article selection process.

### Characteristics of included studies

The characteristics of the seven included studies are outlined in Table 1. Included studies came from five countries (South Africa, Sudan, Nigeria, Senegal, and Kenya), and all studies were conducted among hospitalised patients or patients presenting to the emergency department. Both prospective and retrospective studies were included. The included articles assessed the prevalence of AMI among very different study populations, such as patients admitted to the hospital with a diagnosis of acute stroke, patients with diabetes who presented to the emergency department, and intensive care unit patients.[17], [21], [22] The number of study patients ranged from 67 to 77,429 patients, and the mean age of study participants ranged from 55.6 to 63.9 years. In two studies, objective testing for AMI (such as EKG or cardiac biomarkers) was performed on all participants regardless of clinical presentation; in the other five studies, objective testing was performed at the discretion of the clinician treating the patient.

**Table 1:** Studies characteristics.

Author (Year)	Country	Study design	Population	Subjects (N)	Age, mean (years)	% Male	All subjects screened?*
Joubert, et al (2000)	South Africa	Prospective Cross- sectional	Patients admitted for acute stroke	555	56.5	43%	Y
Ahmed, et al (2000)	Sudan	Retrospective Cross- sectional	Diabetic patients who died while in hospital	67	55.8	34%	N
Sani, et al (2006)	Nigeria	Retrospective Cross- sectional	Inpatients on medical ward	5124	60.2	72%	N
Seck, et al (2007)	Senegal	Retrospective Cross- sectional	Patients presenting to the emergency department	77,429	59.4	77%	N
Nguchu, et al (2009)	Kenya	Prospective Cross- sectional	Emergency department patients with diabetes	400	63.3	60%	Y
Shavadia, et al (2012)	Kenya	Prospective Cross- sectional	Intensive care unit and step-down patients	2156	63.9	75%	N

*Indicates whether all subjects were screened with an objective test (EKG or cardiac biomarkers) or if screening was performed only for those patients whom the clinician felt had symptoms concerning for possible AMI.

### Quality of studies

**Figure 2:**
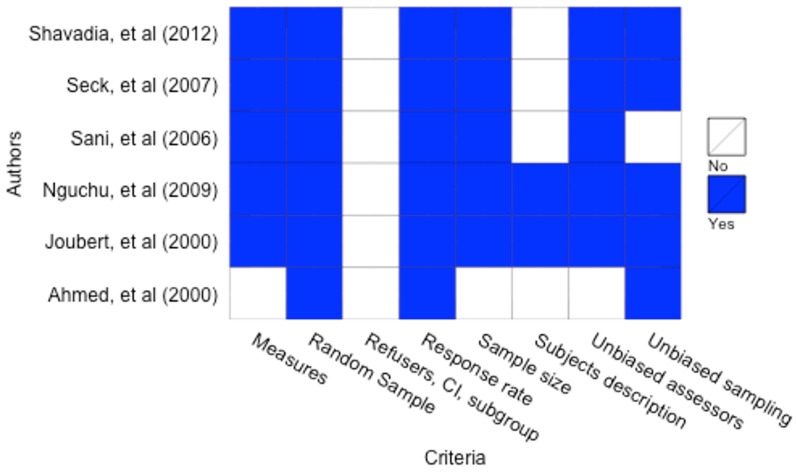
Methodological quality assessment of included studies.*Blue cells indicate a score of “1” (present) and white cells indicate a score of “0” (absent).

The results of the quality assessment of the included studies using the Loney score are demonstrated in Figure 2. Quality scores of the included studies ranged from 3 to 7 on an 8-point scale, with a score of 8 indicating highest quality. Two studies demonstrated particularly higher methodological quality.[17], [21] All included studies reported data from the entire population under study; no randomization techniques were used. None of the included studies reported a participant response rate or refusal rate.

### Synthesis of results

**Figure 3:**
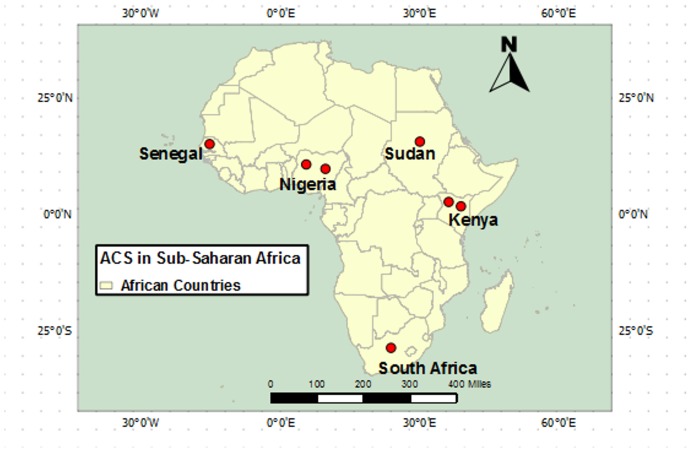
Locations of studies of AMI in Sub-Populations of Sub-Saharan Africa.

The prevalence of AMI among each study population is presented in Table 2. The prevalence of AMI ranged from 0.1% in Senegal among patients presenting to an emergency department, to 10.4% in Sudan among diabetic patients who died while in hospital.[18], [20] Due to heterogeneity of study populations, we did not assess for temporal trends. Figure 3 demonstrates the geographic distribution of included studies and their respective AMI prevalences.

**Table 2:** Prevalence of MI among study populations of included studies.

Author (Year)	Country	Population	Subjects with MI (n)	Total Subjects (N)	Prevalence of MI (%)
Joubert, et al (2000)[17]	South Africa	Patients admitted for acute stroke	4	555	0.7%
Ahmed, et al (2000)[18]	Sudan	Diabetic patients who died while in hospital	7	67	10.4%
Sani, et al (2006)[19]	Nigeria	Inpatients on medical ward	22	5124	0.4%
Seck, et al (2007)[20]	Senegal	Emergency department patients	52	77,429	0.1%
Nguchu, et al (2009)[21]	Kenya	Emergency department patients with diabetes	10	400	2.5%
Shavadia, et al (2012)[22]	Kenya	Intensive care unit and step-down patients	62	2156	2.9%
Kolo, et al (2013)[16]	Nigeria	Inpatients on medical ward	14	6647	0.2%

## Discussion

Despite inclusive criteria and an exhaustive search strategy including more than 2000 studies, only seven studies reporting the prevalence of AMI in Sub-Saharan countries were found. Given the absence of high-quality studies of the prevalence of AMI in SSA, the paucity of studies reporting the prevalence of AMI even among narrowly defined sub-populations in the region highlights the urgent need for more data.[9] The two studies with the largest and most broadly defined study populations were conducted among all patients who presented to an emergency department in Senegal and all medical inpatients at a hospital in Nigeria.[16], [20] These studies also reported the lowest prevalence of MI (0.1% and 0.2%, respectively) of all included studies, although it is impossible to determine whether other cases were missed because of the studies' limited discussions of their review methods. Studies conducted among more narrowly defined populations with risk factors for coronary artery disease, such as diabetic patients who died while in a hospital in Sudan, reported prevalences of AMI that were orders of magnitude larger (10.4%).[18] Given the heterogeneity among study populations, it is impossible to make geographic comparisons of AMI prevalence across the continent or to determine whether the prevalence of AMI has been increasing in the face of rapid growth of risk factors for ischemic heart disease. High-quality studies conducted in community-wide populations in different countries and spanning multiple years are needed to allow for these kinds of analyses.

In spite of these limitations, the evidence reviewed suggests that the prevalence of AMI in SSA remains lower than the prevalence in wealthier countries, including in hospitalized and critically ill patients. For instance, the risk of hospital admission for AMI among Medicare beneficiaries in the United States from 2000–2006 was 1.40% per beneficiary per year.[23] Moreover, according to the 2006 United States National Hospital Discharge Survey, 4.5% of all patients admitted to acute care hospitals for non-surgical reasons were diagnosed with ischemic heart disease (IHD) on that admission, and 16·6% of patients discharged had been given a diagnosis of IHD at some point in their lives.[24] For higher-acuity patients, IHD was the admitting diagnosis in 16.1% of all United States ICU admissions in 1989.[25] Among Swiss stroke patients in particular, the Lausanne Stroke Registry documented a history of ischemic heart disease in 21.7% of patients.[26] These prevalences are substantially higher than the prevalences found in the few studies conducted among similar populations in SSA reviewed here.

The apparent difference in AMI prevalence in SSA and wealthy countries may be due to a lower prevalence of risk factors for cardiovascular disease in SSA, the relatively higher burden of infectious disease in SSA, different patterns of care if patients with chest pain in SSA are less likely to end up in hospital settings, or systemic biases if cases of AMI were missed by the clinicians in those studies included in this analysis in which the clinician decided whether or not to screen each subject with an EKG or cardiac biomarkers. The possibility of missed diagnosis of AMI by local clinicians is particularly important to consider as anecdotal evidence suggests that local providers may misattribute anginal symptoms to infectious causes. Evidence from wealthy nations suggests that misdiagnosis in fatal conditions occurs at least 14% of the time.[27] Indeed, given the scarcity of high-quality data regarding the prevalence of ischemic heart disease in SSA, providers may misdiagnose cases of AMI because they believe the disease to be uncommon.[28] Such misdiagnosis could lead to underestimation of disease prevalence from death registries, thereby strengthening misperceptions of disease rarity and spawning a cycle of misdiagnosis and neglect of AMI. The apparent difference between AMI prevalence among similar populations in the West and SSA may also be due to protective genetic factors against cardiovascular disease among sub-Saharan Africans. The possibility of protective genetic factors or inflammatory profiles has been raised by other researchers, but such lines of argument remain purely speculative in the absence of higher-quality data regarding AMI prevalence in the sub-continent.[29]

Our analysis illustrates that we continue to know very little about the evolving incidence of AMI in SSA, and it should galvanize efforts to generate reliable statistics and plan future funding of cardiovascular disease treatment and prevention. International donors must consider the generation of accurate disease statistics in their support of ministries of health. Some efforts are already being made to collect these statistics, such as the Registry for Acute Coronary Events in Nigeria (RACE Nigeria), which aims to enroll acute coronary syndrome patients in the catchment areas of several major Nigerian medical centers.[30] The upcoming publication of the 2013 Global Burden of Disease Study (GBD) will likely continue to use mathematical modeling to estimate the prevalence of ischemic heart disease in sub-Saharan Africa. The studies included here complement the GBD by highlighting the paucity of data from SSA and the limited distribution of current data sources, as well as providing more detailed prevalence estimates for AMI.

Although the generation of consistent billing codes or standardized public health reporting to monitor epidemiologic trends in AMI is currently not feasible across the whole subcontinent, an initial hospital discharge survey using universal screening and standardized diagnoses and administered via mobile phone or similar technology in select representative regions may prove to be the most economical way to generate such data. Mobile phone technology has already proven effective in infectious disease surveillance in Uganda and Sri Lanka.[31] These data are critical to safeguard the health of the next generation in SSA. The near future may be our last opportunity to track and prepare for an increase in chronic cardiovascular disease before SSA must add yet another epidemic to its already-formidable burden of morbidity.

### Limitations

There were several limitations to our review. Firstly, nearly all of the studies reviewed were conducted in specific subpopulations, such as acute stroke patients and diabetic patients presenting to the emergency department, making inferences about the prevalence of AMI in the general population difficult. Secondly, by requiring either enzymes or EKG in the diagnosis of presumed AMI, we excluded several studies that diagnosed AMI based on clinical exam alone. This search strategy may have introduced bias by favoring studies conducted in medical facilities in which advanced diagnostic equipment was available and patients with more comorbidities present, resulting in potential overestimation of the population prevalence of ACS and limiting the applicability of study findings to the general population. However, we felt the accuracy of diagnosis was important and required some objective measure to be valid. Thirdly, nearly all of the included studies were conducted in urban centers, likely representing a publication bias and disproportionately sampling patients with unique behavioral and socioeconomic risk factors for coronary artery disease. The findings of these studies, therefore, would have little applicability to poorer, rural communities.

Finally, five of the included studies were subject to reporting bias as IHD was diagnosed only if the treating clinician initiated a workup for it, including EKGs and cardiac biomarkers.[16], [18]-[20], [22] As previously discussed, some clinicians might not explore potential angina symptoms with a relatively expensive cardiac workup. It is unknown what proportion of these patients present with atypical symptoms as compared with wealthier nations, since most patients with atypical symptoms would likely be missed in areas of low disease incidence. Furthermore, in a region in which emergency services and community education on AMI symptoms are often absent, patients may disregard the symptoms of AMI or fail to obtain transport to an emergency department until they are in cardiac arrest, leading to an incalculable number of unreported pre-hospital deaths due to AMI. Given the limitations in the above mentioned studies, we elected not to conduct this review in accordance with Cochrane Collaborative guidelines as we anticipated that none of the above articles would meet strict Cochrane criteria.

## Conclusions

We conclude that the reported prevalence of AMI remains low in SSA by recent studies, which are limited by incompletely described methodologies. Current data quality does not allow for analysis of temporal or geographical trends. Further studies of AMI in SSA are sorely needed in order to guide future prevention and treatment strategies on a population basis.

## Supporting Information

Checklist S1 **PRISMA Guidelines.** Checklist showing locations in text for the Preferred Reporting Items for Systematic Reviews and Meta-Analyses (PRISMA).
(DOCX)

Appendix S1 **Search Strategy.** Search strategy used in PubMed.
(DOCX)
