# Supplementary material for: Acute Myocardial Infarction in Sub-Saharan Africa: The Need for Data
Source: PLoS One. 2014 May 9;9(5):e96688. doi: 10.1371/journal.pone.0096688 (PMC4016044; doi:10.1371/journal.pone.0096688)
Supplement: Appendix S1 — Search Strategy. Search strategy used in PubMed. (DOCX) [file pone.0096688.s002.docx]

**Appendix S1: Search Strategy.**

Search strategy used for PubMed:

| **Search** | **Query** | **Items found** |
| --- | --- | --- |
| #3 | Search #1 AND #2 | 1485 |
| #2 | Search "Africa" [Mesh] OR "Africa South of the Sahara" [Mesh] OR Subsaharan Africa [All Terms] OR Africa, Sub-Saharan [All Terms] OR Sub-Saharan Africa [All Terms] OR Sub-Sahar* [All Terms] OR "Africa, Central" [Mesh] OR Central Africa [All Terms] OR Africa, Eastern [Mesh] OR East* Africa [All Terms] OR "Africa, Western" [Mesh] OR West* Africa [All Terms] OR "Africa, Southern" [Mesh] OR South* Africa [All Terms] | 201216 |
| #1 | Search "Chest Pain" [Mesh] OR Chest Pains [All Terms] OR Pain, Chest [All Terms] OR Pains, Chest [All Terms] OR "Acute Coronary Syndrome" [Mesh] OR Acute Coronary Syndromes [All Terms] OR Coronary Syndrome, Acute [All Terms] OR Coronary Syndromes, Acute [All Terms] OR Syndrome, Acute Coronary [All Terms] OR Syndromes, Acute Coronary [All Terms] OR "Myocardial Ischemia" [Mesh] OR Ischemia, Myocardial [All Terms] OR Ischemias, Myocardial [All Terms] OR Myocardial Ischemias [All Terms] OR Ischemic Heart Disease [All Terms] OR Heart Disease, Ischemic [All Terms] OR Disease, Ischemic Heart [All Terms] OR Diseases, Ischemic Heart [All Terms] OR Heart Diseases, Ischemic [All Terms] OR Ischemic Heart Diseases [All Terms] OR "Angina Pectoris" [Mesh] OR "Angina, Unstable" [Mesh] OR Anginas, Unstable [All Terms] OR Unstable Anginas [All Terms] OR Angina, Preinfarction [All Terms] OR Anginas, Preinfarction [All Terms] OR Preinfarction Angina [All Terms] OR Preinfarction Anginas [All Terms] OR Angina Pectoris, Unstable [All Terms] OR Angina Pectori, Unstable [All Terms] OR Unstable Angina Pectori [All Terms] OR Unstable Angina Pectoris [All Terms] OR Unstable Angina [All Terms] OR Angina at Rest [All Terms] OR Myocardial Preinfarction Syndrome [All Terms] OR Myocardial Preinfarction Syndromes [All Terms] OR Preinfarction Syndrome, Myocardial [All Terms] OR Preinfarction Syndromes, Myocardial [All Terms] OR Syndrome, Myocardial Preinfarction [All Terms] OR Syndromes, Myocardial Preinfarction [All Terms] OR "Myocardial Infarction" [Mesh] OR Infarction, Myocardial [All Terms] OR Infarctions, Myocardial [All Terms] OR Myocardial Infarctions [All Terms] OR Myocardial Infarct [All Terms] OR Infarct, Myocardial [All Terms] OR Infarcts, Myocardial [All Terms] OR Myocardial Infarcts [All Terms] | 410711 |
